# Resolution of Trifascicular Heart Block with Effective Closure of Congenital Atrial Septal Defect Followed by Later Coronavirus Disease 2019–associated Cardiac Strain

**DOI:** 10.19102/icrm.2023.14081

**Published:** 2023-08-15

**Authors:** Yasser Mohammed Hassanain Elsayed, Alsayed Ali Almarghany

**Affiliations:** ^1^Department of Critical Care, Egyptian Ministry of Health (MOH), Damietta Health Affairs, Damietta, Egypt; ^2^Department of Cardiology, Faculty of Medicine, Al-Azhar University, Cairo, Egypt

**Keywords:** Atrial septal defect, cardiac strain, congenital heart disease, COVID-19, trifascicular block

## Abstract

Heart block (HB) is one of the most serious arrhythmias. Higher degrees of HB—for example, trifascicular HB—result in a more intense patient condition. Atrial septal defects (ASDs) represent the most common congenital heart disease in adults. All ASDs generally result in a left-to-right shunt, commonly causing right-side enlargement and dilation and, to a lesser extent, left atrial enlargement. A 26-year-old woman presented to the physician outpatient clinic with a complicated ASD with trifascicular HB and severe mitral and tricuspid regurgitations. The trifascicular HB with valvular regurgitations resolved with congenital ASD closure; however, she was diagnosed with coronavirus disease 2019 (COVID-19)–associated cardiac strain 3 years later. Interventions included electrocardiography, oxygenation, echocardiography, and cardiovascular surgical repair. A dramatic electrocardiographic response and better clinical outcomes despite dilations of both atria were observed. Trifascicular HB is a newly recorded association after congenital ASDs in adults. The disappearance of trifascicular HB after surgical closure of the congenital ASD is an indicator of effective surgical repair. The occurrence of COVID-19 pneumonia later, with atrial dilations continuing after the infection, may be a constellation of risk factors for the observed cardiac strain.

## Introduction

Atrial septal defect (ASD) is one of the most common types of congenital heart defects.^1^ Statistical data suggest that ASDs occur in 1.6 per 1,000 live births.^2^ ASD is one of the most familiar and common congenital cardiac anomalies in adults.^3^ ASD accounts for 10% of all congenital heart diseases (CHDs) and 22%–40% of CHDs in adults. The incidence of ASD in girls is twice that in boys.^3^ An ASD occurs when there is a failure to close the communication between the right and left atria.^1^ Patent foramen ovale, ostium secundum defect, ostium primum defect, sinus venosus defect, and coronary sinus defect are 5 known types of ASDs.^1^ An ostium secundum defect is the most frequent type of ASD (75% of all cases) and exists in the area of the fossa ovalis. The second-most common type of ASD is an ostium primum defect, which represents 15% of ASDs and occurs in the lower part of the interatrial septum. It can be associated with a “cleft” anterior mitral valve leaflet, which causes mitral regurgitation (MR) or atrioventricular (AV) or membranous interventricular septum defects.^3^

Electrocardiography (ECG) may be helpful as a diagnostic modality in the assessment of ASD. Associated ECG abnormalities with ASD can include right atrial enlargement, right-axis deviation (RAD), P–R prolongation (or first-degree heart block [HB]), right bundle branch block (RBBB), right ventricular hypertrophy, left-axis deviation (LAD), atrial fibrillation (AF), atrial flutter, and junctional rhythms.^4,5^ Ostium secundum ASDs usually cause AF or atrial flutter.^4,5^ About 2 out of 3 patients with an ostium secundum ASD have RAD and incomplete RBBB. A first-degree HB is found to occur more frequently with an ostium primum ASD due to the involvement of the His bundle in the proximity of the defect. Both of these can cause an increased distance of internodal conduction from the sinoatrial node to the AV node. Ostium primum ASDs are associated with a marked superior LAD.^4,5^ Transthoracic echocardiography is the imaging test of choice for ASD and helps to assess the size of the defect, understand the direction of blood flow and valve regurgitation, and detect the associated abnormalities.^1^ Small ASDs generally spontaneously close during childhood, while large ASDs typically do not close spontaneously and may require percutaneous or surgical intervention to prevent probable complications, such as stroke, arrhythmias, and pulmonary hypertension (PHT).^1^ Patients with an ASD <5 mm in size frequently experience spontaneous closure of the defect in the first year of life.^6^ Defects that are >1 cm in size will most likely require medical and surgical intervention to achieve closure, although they do carry a weak chance for possible spontaneous closure. The diameter of the ASD and age during diagnosis are the most important predictors of spontaneous closure and the need for possible surgical or device closure.^6^

Trifascicular HB is the combination of a bifascicular block and a first-degree HB. The most important causes of trifascicular HB are ischemic artery disease, anterior myocardial infarction (MI), hypertension, CHD, a primary degenerative disease of the conducting system (such as Lenègre–Lev disease), aortic stenosis, digitalis toxicity, and hyperkalemia.^7,8^ There are 2 types of trifascicular HB: the first is incomplete trifascicular HB, which is characterized by a bifascicular HB with a first-degree HB (MCC), a bifascicular block with a second-degree HB, and RBBB with alternating left anterior fascicular block (LAFB)/left posterior fascicular block (LPFB), while the second is complete trifascicular HB, which is characterized by a bifascicular block with a third-degree HB.^7,8^ Several new mechanisms have been implicated in cardiac damage due to the coronavirus disease 2019 (COVID-19) epidemic. The systemic inflammatory response in severe COVID-19 provokes a high level of cytokines, causing a cytokine release syndrome that can injure multiple tissues and involving the vascular endothelium and cardiac myocytes.^8,9^

## Case presentation

**Figure 1:**
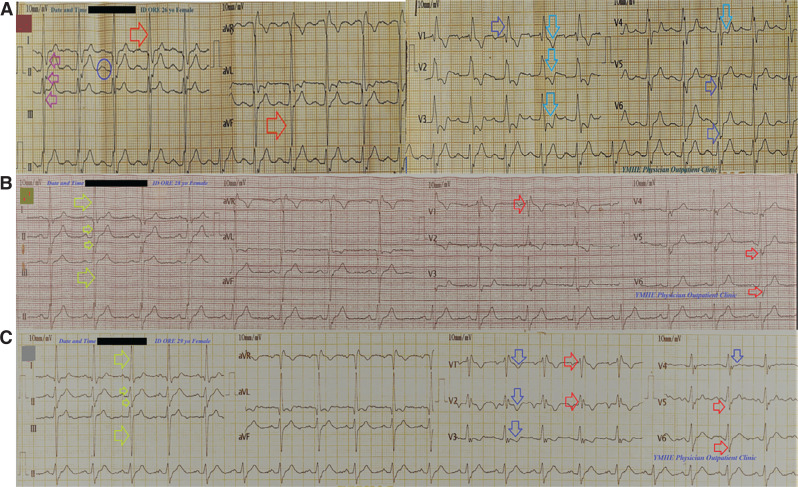
Serial electrocardiogram tracings. **A:** Tracing was done on the day of the presentation to the patient outpatient clinic showing right bundle branch block (RBBB) (dark blue arrows), left-axis deviation (LAD) (orange arrows), prolonged P–R interval (trifascicular heart block; dark blue circle), S1S2S3 pattern (pink arrows), right ventricular (RV) strain (light blue arrows), and a regular rhythm at a ventricular rate (VR) of 89 bpm. **B:** Tracing: postoperative electrocardiography was done within a few days of surgical repairs and showed the disappearance of the trifascicular heart block and RV strain with physiological LAD (lime arrows) and RBBB (red arrows) with a regular rhythm at a VR of 68 bpm. **C:** Electrocardiography was done during coronavirus disease 2019 pneumonia and showed RV strain (dark blue arrows) with physiological LAD (lime arrows) and RBBB (red arrows) with a regular rhythm at a VR of 90 bpm.

**Figure 2:**
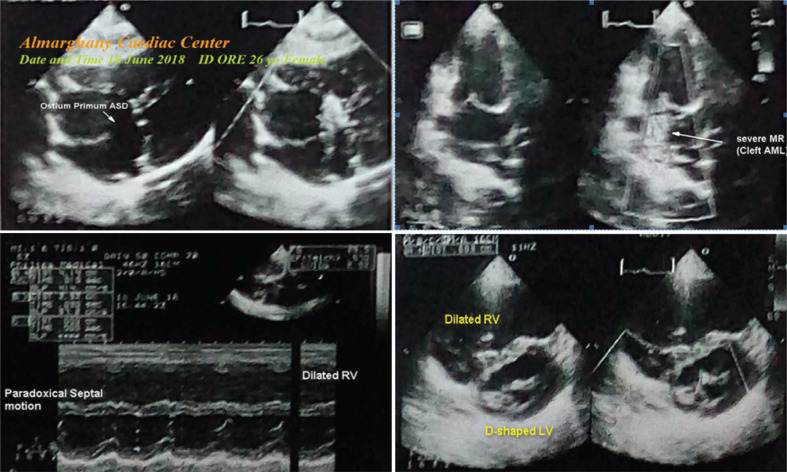
Initial echocardiography was done within 3 days of the patient’s presentation to the outpatient clinic showing an ostium primum atrial septal defect with a left-to-right shunt 2.8 cm in size, severe mitral regurgitation, dilated right ventricle, paradoxical septal motion, tricuspid regurgitation, and mild pulmonary hypertension (47 mmHg) with an ejection fraction of 57%. *Abbreviations:* AML, anterior mitral valve leaflet; ASD, atrial septal defect; LV, left ventricle; MR, mitral regurgitation; RV, right ventricle.

A 26-year-old woman presented to the physician outpatient clinic (POC) with dyspnea, tachypnea, palpitations, and chest pain and reported that these symptoms had progressed over a few weeks. The patient denied a history of other relevant diseases, drugs, or other special habits. During the general physical examination, generally, the patient appeared distressed and tachypneic with dyspnea. She had a regular pulse rate of 95 bpm, a blood pressure of 110/70 mmHg, a respiratory rate of 20 bpm, a temperature of 36.5°C, and a pulse oximeter oxygen (O_2_) saturation of 94%. A soft systolic ejection murmur was heard over the pulmonary area with a wide fixed splitting of S2. Also, a pansystolic murmur was heard in both the mitral and pulmonary areas. Initially, the patient was treated in the POC with O_2_ inhalation using an O_2_ generator (100%, by nasal cannula, 5 L/min). The patient was maintained on a low dose of oral furosemide (40 mg). The emergency ECG showed RBBB, LAD, prolonged P–R interval (trifascicular HB), S1S2S3 pattern, and right ventricular (RV) strain with a regular rhythm at a ventricular rate (VR) of 89 bpm **(Figure 1A)**. Measurements from her first workup (complete blood count, random blood sugar, serum glutamic–pyruvic transaminase, serum glutamic–oxaloacetic transaminase, serum creatinine, blood urea, D-dimer, and troponin) were within normal limits. Initial echocardiography showed an ostium primum ASD with a left-to-right shunt 2.8 cm in size, severe MR and tricuspid regurgitation (TR), and mild PHT (47 mmHg) with an ejection fraction (EF) of 57% **(Figure 2)**. Surgical repair was completed via a median sternotomy. Cardiopulmonary bypass was used for the closure of the defect using a pericardial patch. A postoperative ECG was done within a few days of the surgical repair, which confirmed the disappearance of the trifascicular HB and RV strain with physiological LAD and RBBB with a regular rhythm at a VR of 68 bpm **(Figure 1B)**. Serial yearly echocardiography showed a closed ostium primum ASD with gradual improvement in MR, TR, PHT, and EF; (65%) and, a remaining dilated LA chamber.

The patient suffered an attack of COVID-19 pneumonia within 3 years of closure of the ostium primum ASD. An ECG was done during the COVID-19 pneumonia, which showed RV strain with physiological LAD and RBBB with a regular rhythm at a VR of 90 bpm **(Figure 1C)**. Her COVID-19 pneumonia was treated and improved after the use of traditional medications according to both national and international guidelines. A low dose of captopril (12.5 mg once daily) was the only continued medication.

Overall, the patient showed gradual clinical (7 days), ECG (14 days), and echocardiographic improvements (in a few months) following surgical repair. Generally, trifascicular HB with congenital ASD with valvular regurgitations and later COVID-19–associated cardiac strain were the most probable diagnoses. The follow-up after surgical repair for the patient was daily for about 14 days, then monthly for 6 months, and then twice yearly thereafter, but the follow-up after COVID-19 pneumonia was daily for about 14 days and then monthly for a few months.

Informed consent was obtained from the patient.

## Discussion

### Overview

The patient presented to the POC with a complicated congenital ASD with trifascicular HB and severe MR and TR. The primary objective for this case study was to detail the presence of a complicated ASD with trifascicular HB and severe MR and TR at POC.

Trifascicular HB is a newly reported complication in a congenital ASD. There is no known mechanism.

A congenital ASD with severe MR, TR, and PHT was the main echocardiographic diagnosis in this case. There was a dramatic ECG improvement in the patient’s trifascicular HB with RV strain and axis deviation following surgical closure of the congenital ASD.

The reported ECG changes before and after medication or surgical intervention are considered indirect evidence for the efficacy of this drug or intervention. Elsayed^10^ reported a case of acute MI associated with RBBB and changeable trifascicular block. The accompanying trifascicular HB in this previous case boasted pre-streptokinase LAFB with LAD and post-streptokinase LPFB with RAD. In the current case, a therapeutic test can be applied retrospectively but should be relevant to the effect of surgical repair on the change of the axis from a pathological LAD to a physiological one. The LAD in ostium primum ASD may not be due to LAFB^11^ as a differential diagnosis. However, one cannot apply it as long as there is a positive therapeutic effect for surgical repair. So, there is no need to mention the superior QRS axis in ostium primum ASD.

Despite the fact that co-association of both RBBB and LAFB is inappropriate in ASD pathology, it is due to the relationship between the ECG, ventricular activation, and the ventricular conduction system in ostium primum ASD.^12^ However, this association in the current case is an ECG interpretation, and this cannot be ignored. We believe that it is a new finding or association in ostium primum ASD, even though there are no clear pathogeneses.

Notably, the hallmark of recurrent RV strain occurred during COVID-19 pneumonia.

A low dose of captopril (12.5 mg once daily) was the only continued medication to improve cardiac remodeling. AV blocker medications such as β-blockers and non-dihydropyridine calcium antagonists were avoided given the fear of further complete HB.

We cannot compare the current case with similar cases as there are no similar or known cases with the same management available for comparison.

Due to its higher costs, serial coronary angiography was considered a study limitation for follow-up both after surgical repair and after COVID-19 pneumonia.

## Conclusion and recommendations

Trifascicular HB is a newly recorded association after congenital ASDs in adults. The disappearance of trifascicular HB after surgical closure of the congenital ASD is an indicator of effective surgical repair. The occurrence of COVID-19 pneumonia later, with atrial dilations continuing after the infection, may be a constellation of risk factors for our patient’s recent cardiac strain.
